# Adjusted Morbidity Groups and Intracerebral Haemorrhage: A Retrospective Primary Care Cohort Study

**DOI:** 10.3390/ijerph182413320

**Published:** 2021-12-17

**Authors:** Blanca Lorman-Carbó, Josep Lluis Clua-Espuny, Eulalia Muria-Subirats, Juan Ballesta-Ors, Maria Antònia González-Henares, Meritxell Pallejà-Millán, Francisco M. Martín-Luján

**Affiliations:** 1 Primary Health-Care Centre, Institut Català de la Salut, Primary Care Service (SAP) Terres de l’Ebre, 43500 Tortosa, Spain; blancalormancarbo@gmail.com (B.L.-C.); eumuria@gmail.com (E.M.-S.); juan.ballesta.ors@gmail.com (J.B.-O.); doctoragonzalezhenares@gmail.com (M.A.G.-H.); 2 Biomedicine Doctoral Programme, University Rovira i Virgili, 43201 Reus, Spain; 3 Research Support Unit Tarragona, Institut Universitari d’Investigació en Atenció Primària Jordi Gol (IDIAP JGol), 43202 Reus, Spain; mpalleja@idiapjgol.info (M.P.-M.); paco.martin@urv.cat (F.M.M.-L.); 4 Faculty of Medicine and Health Sciences, University Rovira i Virgili, 43201 Reus, Spain

**Keywords:** cerebral haemorrhage, chronicity, multimorbidity, primary health care

## Abstract

Background: Intracerebral haemorrhage rates are increasing among highly complex, elderly patients. The main objective of this study was to identify modifiable risk factors of intracerebral haemorrhage. Methods: Multicentre, retrospective, community-based cohort study was conducted, including patients in the Adjusted Morbidity Group 4 with no history of intracerebral haemorrhage. Cases were obtained from electronic clinical records of the Catalan Institute of Health and were followed up for five years. The primary outcome was the occurrence of intracerebral haemorrhage during the study period. Demographic, clinical and pharmacological variables were included. Logistic regression analyses were carried out to detect prognostic variables for intracerebral haemorrhage. Results: 4686 subjects were included; 170 (3.6%) suffered an intracerebral haemorrhage (85.8/10,000 person–year [95% CI 85.4 to 86.2]). The HAS-BLED score for intracerebral haemorrhage risk detection obtained the best AUC (0.7) when used in the highest complexity level (cut-off point ≥3). Associated independent risk factors were age ≥80 years, high complexity and use of antiplatelet agents. Conclusions: The Adjusted Morbidity Group 4 is associated with a high risk of intracerebral haemorrhage, particularly for highly complex patients and the use of antiplatelet agents. The risk of bleeding in these patients must be closely monitored.

## 1. Introduction

Intracerebral haemorrhage (ICH) accounts for approximately 80% of haemorrhagic strokes and is the second most common subtype of stroke after the ischemic. ICH still has limited treatment options and is one of the leading global causes of disability and mortality [1]. An overall stabilisation of age-adjusted ICH incidence has been documented during the last 30 years. However, an increase of ICH in the elderly suggests that the cumulative incidence and prevalence are likely to increase with population ageing and the increase in life expectancy [2,3]. Newly updated guidelines from the World Health Organisation, the European Stroke Organisation and the Stroke Alliance aim to further decrease ICH mortality and improve disability outcomes [4,5,6]. However, they lack specific guidance for the identification and control of predisposing factors. Population ageing and widespread use of antithrombotic medications demand specific risk stratification strategies to mitigate the modifiable risk factors of ICH in order to reduce morbidity and mortality [2].

Several risk scores to predict bleeding in patients with atrial fibrillation (AF) have been proposed [7,8,9]. However, the score most frequently included in the guidelines is the HAS-BLED, the only one that predicts ICH in patients with AF treated with anticoagulants [10,11]. This score includes the most common bleeding risk factors [12] and is easily implemented in daily practice to identify and follow-up patients with and without AF [13]. Recent studies from our setting show that complex chronic patients are a high-risk group for ICH, with ICH incidences from 5 to 60 times higher than the general population. Notably, the HAS-BLED has shown high sensitivity in this subgroup of population for the identification of individuals at high risk of ICH [14,15].

Complexity reflects the difficulty of managing a patient’s care requirements and the need to apply specific individual care plans that take into account multimorbidity, health services use and the patient’s environment [16]. Approximately 4–5% of the Spanish population are considered complex patients [16]. Since the definition of complexity can be subjective, it is essential to use stratification models based on comorbidities to identify subpopulations of interest in terms of mortality, hospitalisation, primary care attendance and pharmaceutical consumption [17]. The Adjusted Morbidity Groups (GMA in its Spanish acronym) are a novel tool for population grouping and risk stratification; they have been developed with data from the Spanish health systems and have obtained good results when compared with other European risk assessment and stratification strategies [18,19,20]. GMAs are currently part of the Chronic Care Strategy of the Spanish Ministry of Health, and over 80% of the Spanish population has already been stratified in GMA [21]. Supplementary S1 of Supplementary Materials details the variables of the GMA system. Essentially, the GMA considers the type of disease (acute or chronic), the number of systems affected and the complexity of each disease in order to classify people in four strata based on their morbidity-associated risk (from mildest (GMA-1) to most severe (GMA-4)). 

Understanding ICH in highly complex patients is essential to implement preventative strategies and improve management. The aim of this study is to evaluate the risk of ICH among the GMA-4 population and to identify potentially modifiable risk factors.

## 2. Materials and Methods

### 2.1. Study Design

This is a multicentre, retrospective, community-based cohort study including patients in the most severe morbidity adjusted group (GMA-4), followed up for five years (from 1 January 2015 to 31 December 2019) in primary care centres in the Terres de l’Ebre health area, Catalonia, Spain. This geographical area includes eleven primary care teams operating collaboratively with a hospital with secondary care services, a nursing home, mental health and social services. This model aims to manage the enhanced needs and demands generated by patients with frequent exacerbations and intensive use of healthcare services.

### 2.2. Patients

All patients included in this study corresponded to the most severe morbidity adjusted group (GMA-4) with no prior history of ICH at the beginning of the study and had an active medical record in one of the participating health centres. The clinical record system automatically defines the risk status of patients according to GMA criteria. The patients had to live in the study area, including long-term nursing/residential care facilities.

The exclusion criteria were: (1) diagnosis of progressive and irreversible chronic disease unlikely to respond to specific treatments and with limited life prognosis (MACA, its Catalan acronym for Advanced Chronic Care Model) [16]; (2) pregnancy; and (3) patients with a history of cancer or active cancer. Active oncological disease and pregnancy and/or childbirth are differentiated in the GMA stratification and excluded from the GMA-4 subgroup (see Supplementary S2 of Supplementary Materials). 

### 2.3. Outcomes

The main outcome was the diagnosis of an ICH episode (ICD-10 code I60–70) during the study period. The follow-up time was established from the registration as GMA-4 in the clinical records (1 January 2015) until the end of the study, the occurrence of an ICH event or death from any cause. 

### 2.4. Covariates

In addition to socio-demographic covariates (age, sex and type of residence (considered institutionalised if they lived in a long-term nursing/residential care home)), the main covariates for this study were clinical and pharmacological.

#### 2.4.1. Categorical Variables

Hypertension, diabetes mellitus, hypercholesterolemia, atrial fibrillation, heart failure, coronary artery disease, stroke or transient ischemic attack, peripheral arterial disease, thromboembolism, chronic kidney disease, chronic liver disease, cognitive impairment or dementia, record of previous falls and smoking. The variable cardiovascular disease was created, including the following diagnoses: ischemic heart disease, stroke or transient ischemic attack and/or peripheral arterial disease. Complexity was categorised as low (levels 2 and 3) and high (levels 4 and 5). ICD-10 was used to code all clinical diagnoses. 

#### 2.4.2. Continuous Variables

Mean systolic blood pressure (SBP; in mmHg), glycosylated haemoglobin A1c (%) and the variables contained in the HAS-BLED scale: elderly (age > 65 years); uncontrolled hypertension (SBP ≥ 160 mmHg); abnormal liver function (cirrhosis or bilirubin > 2× normal values with AST/ALT/AP > 3× normal values); abnormal kidney function (dialysis, transplant, Cr > 2.3 mg/dL [or > 200 µmol/L]); history of stroke; bleeding tendency or predisposition; labile INRs (unstable/high value, time in therapeutic range < 60%) in patients taking vitamin K antagonists (VKA); use of antiplatelet agents or nonsteroidal anti-inflammatory drug (NSAIDs); and high-risk alcohol consumption (≥8 drinks/week). A HAS-BLED score ≥ 3 indicates an increased risk of bleeding [12].

Pharmacological treatments: oral anticoagulants including VKA and non-vitamin K antagonist oral anticoagulants (NOACs), antiplatelet agents, NSAIDs, selective serotonin reuptake inhibitors (SSRIs), statins and proton pump inhibitors (PPI). All pharmacological variables were coded according to the Anatomical Therapeutic Chemical Classification System (ATC code).

### 2.5. Data Source

The Department of Information and New Technologies of the Management Department of Terres de l’Ebre (Catalan Institute of Health) performed an automated extraction of the data, which were included in an ad hoc repository. All data were considered confidential and treated according to Regulation 2016/679 of April 27 of the European Parliament and Council on Data Protection and the Spanish Organic Law 3/2018 of December 5. The GMA-4 group was automatically identified from the e-SAP database of the Catalan Institute of Health, as well as the demographic and clinical ICD-10 codes. Pharmacological variables were collected from the SIRE (Catalan acronym for Integrated Electronic Prescription System). In addition, the HAS-BLED score was automatically calculated for all patients based on the data registered in their medical history at the beginning of the study.

### 2.6. Statistical Analysis

Data are presented using frequencies and percentages for categorical variables, means with standard deviations for continuous variables, and median and first and third quartiles (interquartile range [IQR]) for not normally distributed variables. To detect differences between the two groups, we used the χ^2^ test for categorical variables and the *t*-test or Mann–Whitney U-test for continuous variables depending on whether the variables were normally distributed or not (respectively), as indicated by the Shapiro–Wilk test.

The total incidence density (ID) of ICH was calculated by 10,000 person–years and stratified by age groups. Multiple linear regression models were used to assess the association between ICH risk and complexity, considering ICH as a response variable and high or low GMA complexity as a factor of the study. Variables associated with a higher risk of ICH based on available evidence and clinical significance were included in the analysis (socio-demographic, cardiovascular risk factors, comorbidities, clinical data and pharmacological variables) [22,23]. To select the final model, a stepwise algorithm was performed in both directions, and a model was chosen according to the minimal value of Akaike Information Criterion (AIC) and clinical relevance. The results are presented as odds ratio (OR) with 95% confidence intervals (CIs).

Cox proportional-hazards regression models were used to estimate the hazard ratio (HR) and 95% CIs, with time until ICH as response variable. With the same variables of the logistic regression model, we performed a non-adjusted model, a model adjusted by sex and age, and a multivariate adjusted model. Survival Kaplan–Meier curves for each GMA-complexity group (high/low) were plotted for ICH incidence in the entire study population and mortality in the ICH group.

The statistical package R (R Foundation for Statistical Computing, Vienna, Austria; version R 3.4.3 for Windows) was used for all analyses. Statistical significance was set at *p*-value < 0.05.

## 3. Results

### 3.1. Characteristics of the Study Population

**Figure 1:**
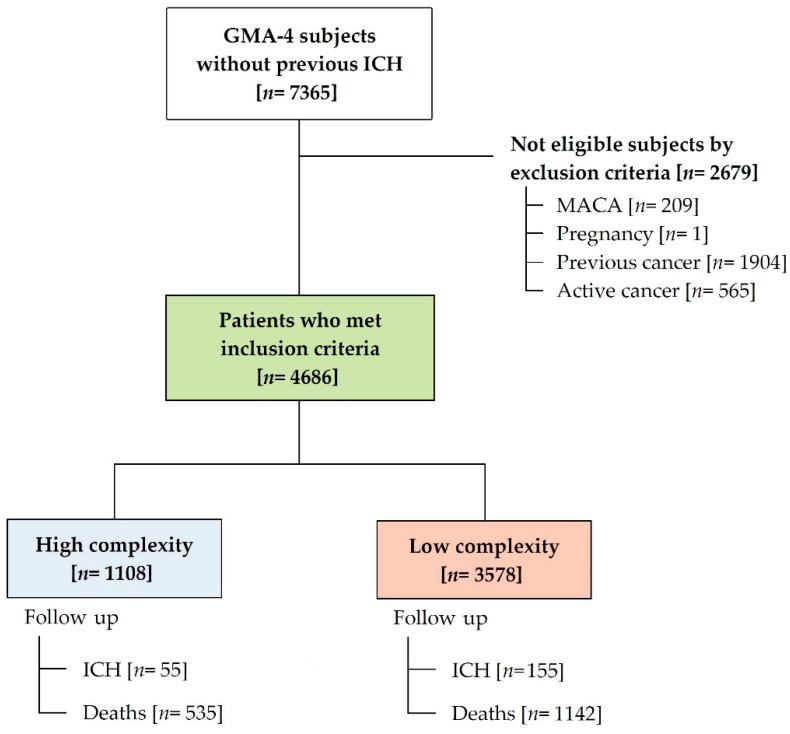
Flow diagram of the study: participant selection (included and excluded), distribution in groups and follow-up according to ICH occurrence. ICH—intracerebral haemorrhage; MACA (Catalan acronym for Advanced Chronic Care Model)—diagnosis of progressive and irreversible chronic disease unlikely to respond to specific treatments and with a limited life prognosis.

In total, 4.83% of the adult population (from a total of 152,351 people 15 and over) in the Terres de l’Ebre was registered as GMA-4. After excluding 2679 subjects due to ineligibility, 4686 GMA-4 subjects (1108 highly complex patients) were preselected. A total of 170 (3.6%) ICH events were registered during the study period (ID of 85.8/10,000 person-year (95%CI from 85.4 to 86.2)), of which 55 (32.4%) happened among the high-complexity subgroup. Figure 1 shows a flow diagram of the study.

Out of 4,686 participants analysed, 57.2% were women, and the median age was 84 years (IQR from 75 to 90); 93% of the population was ≥65 years old, and 64.3% were ≥80 years old. Follow-up time was 5 years (IQR from 3.7 to 5.0), differing between patients who suffered an ICH event and patients with no ICH (ICH 2.6 years (IQR from 1.3 to 3.6) vs. no-ICH 5 years (IQR from 4.0 to 5.0), *p* < 0.001). Table 1 shows the baseline characteristics of the study population according to ICH occurrence.

Relevant differences were found in patients with ICH compared to patients without ICH with respect to the following factors: higher percentage of ≥80-year-old patients (72.9% vs. 64.0%; *p* = 0.021), higher prevalence of cardiovascular disease (39.4% vs. 30.4%; *p* = 0.015), higher prevalence of history of ischemic stroke or transient ischemic attack (11.8% vs. 6.8%; *p* = 0.018); and higher use of antiplatelet agents (66.5% vs. 54.9%; *p* = 0.004) and NOACs (5.9% vs 2.6%; *p* = 0.025). No significant differences in the HAS-BLED score were found. 

Interestingly, we observed differences by sex in patients who suffered an ICH episode (see Table 2). ICH density incidence in men was 94.7/10,000 person–year (95% CI from 94.4 to 94.9), and 79.3/10,000 person–year in women (95% CI from 79.1 to 79.5). Women were older than men (87 years (IQR from 82 to 92) vs. 82 years (IQR from 77 to 88); *p* < 0.001), had a higher prevalence of hypercholesterolemia (63.7% vs. 46.8%; *p* = 0.040) and a higher use of selective serotonin reuptake inhibitors (49.5% vs. 32.9%; *p* = 0.043). In contrast, men presented a higher prevalence of cardiovascular disease (48.1% vs. 31.9%; *p* = 0.045), diabetes mellitus (53.2% vs. 35.2%; *p* = 0.027) with higher HbA1c (7.0 (IQR from 5.6 to 7.8) vs. 6.2 (IQR from 5.4 to 7.2); *p* = 0.032), a significantly higher high-risk alcohol consumption (34.2% vs. 3.3%; *p* <0.001), and smoking (31.6% vs. 4.4%; *p* <0.001). More men than women scored ≥3 in the HAS-BLED scale (65.6% vs. 32.8%, respectively; *p* <0.001).

A total of 96.2% of the GMA-4 population had four or more chronic diseases, without significant differences between patients who suffered an ICH vs. patients with no ICH; 23.6% of the GMA-4 population were highly complex patients (levels 4 and 5), increasing to 32.4% in patients who suffered an ICH (*p* = 0.037) (see Table 1). The high-complexity subgroup presented a higher density incidence of ICH (129.0/10,000 person–years (95% CI from 128.6 to 129.3)). Significant differences were detected regarding the prevalence of most studied variables (see Table 3), including a higher number of subjects with a HAS-BLED score ≥ 3 (54.8% vs. 40.8%; *p* < 0.001) and higher mortality (49.6% vs. 31.9%; *p* < 0.001). 

### 3.2. ICH Incidence Rates

Table 4 shows the ICH incidence density of highly complex patients stratified by age groups and the ICH ID by HAS-BLED score. The overall ICH ID among the highly complex patients was 129.0/10,000 person-year (95% CI from 28.6 to 129.3). ICH ID reached the highest value in patients over 85 years (138.3/10,000 person–year (95% CI from 137.8 to 138.8)). A high proportion of GMA-4 subjects were also over 85 years of age.

Although not statistically significant, a HAS-BLED score ≥ 3 was associated with an increased ICH risk in the GMA-4 population (OR 1.2 (95% CI from 0.9 to 1.7)). Regarding the validity of the HAS-BLED score to detect ICH risk, the best AUC (0.7) was obtained for GMA-4 subjects with the highest complexity level (5), with a cut-off point of ≥3.

### 3.3. ICH Predictive Factors

High-complexity was found to be a predictive factor of ICH in an unadjusted logistic regression analysis (OR 1.57 (95% IC from 1.13 to 2.18; *p* = 0.007)), and an independent predictive factor in the multivariate analysis (OR 1.42 (95% IC from 1.02 to 1.99; *p* = 0.037)). Table 5 shows the results of the multivariate-adjusted model, including male sex, age ≥ 80 years and treatment with antiplatelet agents as independent predictive factors.

**Figure 2:**
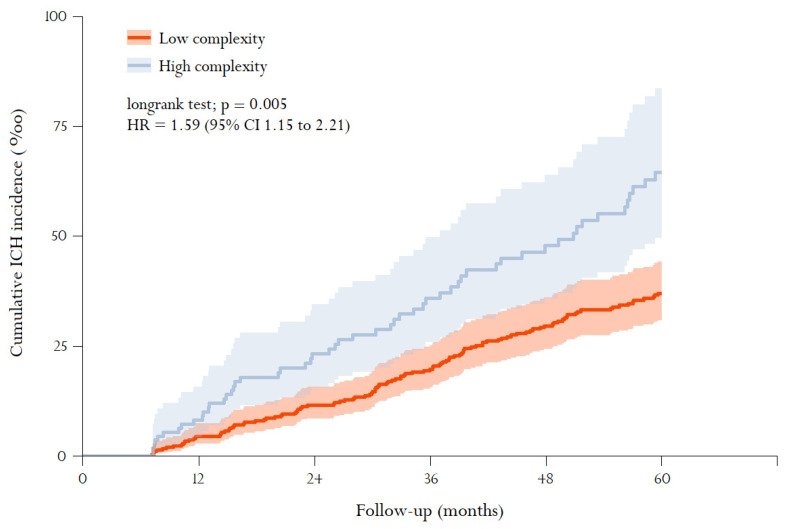
Cumulative ICH incidence according to GMA-complexity group. Kaplan–Meier survival analysis stratified by complexity group (categorised low [levels 2–3] and high [levels 4–5]). The graph shows the cumulative incidence of intracerebral haemorrhage (ICH), calculated as the number of cases that appeared during follow-up (60 months) divided by the number of patients who were disease-free at baseline (‰), with 95% confidence intervals (CI). The bold line corresponds to the value of the accumulated incidence, while the shaded area represents 95% CIs. The log-rank test is used to analyse statistical differences between the survival curves. Cox regression analysis assessing multivariate-adjusted variables: sex, age (categorised ≥ 80 years), GMA complexity (Spanish acronym for Adjusted Morbidity Group), stroke or transient ischemic attack and use of pharmacological treatment (antiplatelet agents and selective serotonin reuptake inhibitors). Data are presented as hazard ratio (HR) and 95%CI for each GMA-complexity group (high/low) in the adjusted model.

Figure 2 shows the comparison of the ICH cumulative incidence curves for each complexity group (high/low). ICH incidence during follow-up was significantly higher in the high-complexity subgroup, with an overall HR of 1.78 (95% CI from 1.29 to 2.45; *p* < 0.01) and 1.59 (95% CI from 1.15 to 2.20; *p* < 0.01) in the multivariate analysis. Other significant variables were age ≥ 80 years (HR 1.77 (95% CI from 1.15 to 2.20; *p* < 0.01)) and treatment with antiplatelet agents (HR 1.48 (95% CI from 1.07 to 2.05; *p* = 0.02)).

### 3.4. Mortality

**Figure 3:**
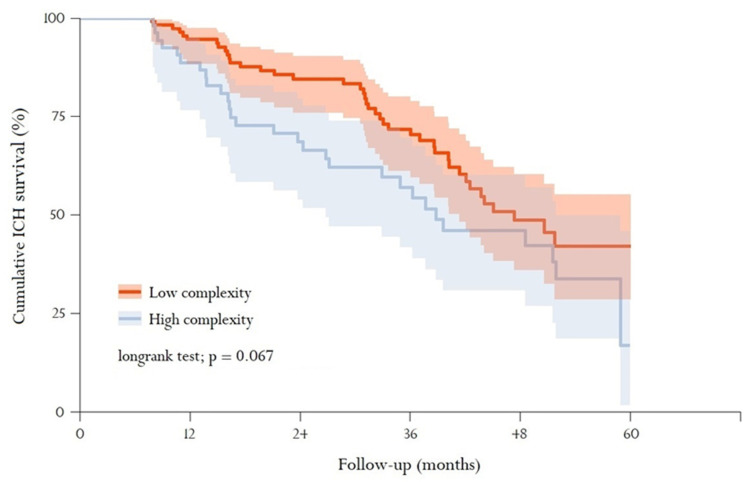
Cumulative survival after an ICH event according to GMA-complexity group. Kaplan–Meier survival analysis stratified by GMA complexity (Spanish acronym for Adjusted Morbidity Group). The graph shows the percentage of patients who suffered an intracerebral haemorrhage (ICH) and survived over time, categorised by low GMA complexity (levels 2–3) and high GMA complexity (levels 4–5). The bold line corresponds to the survival percentage, while the shaded area represents a 95% confidence interval. The log-rank test is used to analyse statistical differences between the survival curves.

Overall mortality was 30.0%, and significantly higher in the ICH group (41.2% vs. 29.5%; *p* = 0.002). Although mortality was higher in highly complex patients compared to patients with low complexity (43.1% vs. 25.9%; *p* <0.001), differences in mortality between groups were not significant when an ICH occurred (52.7 vs. 35.7%, respectively; *p* = 0.051) nor during the follow-up (log-rank test = 0.067) in the ICH group (see Figure 3). No differences in mortality were detected when comparing men with women (43.0 vs. 39.6; *p* = 0.762).

## 4. Discussion

This study introduces the GMA-4 population as a new subgroup at high risk of ICH and provides novel data regarding ICH epidemiology and risk factors. The results underscore the need to review the risk of bleeding according to patient complexity and to drug prescription and emphasise the role of the HAS-BLED score to help reduce the risk of ICH in the GMA-4 population. 

According to the GMA stratification, 4.8% of the adult population in the Terres de l’Ebre were included in the most severe strata (GMA-4), similarly to the estimated 5% in the Spanish population [19]. These patients have more comorbidities, polypharmacy and higher use of health and social services. They also are at greater risk of complications and loss of functional capacity, quality of life and/or early death [19]. Our study found that the GMA-4 population are at high risk of ICH, with a higher ICH incidence density than the general population (85.8/10,000 person–year (95% CI from 85.4 to 86.2) vs. 24.7/10,000 person–years (95% CI from 20.4 to 29.9)) [24]. Based on these results and according to the 2020 Catalan population census, more than 3500 GMA-4 patients over 60 years of age suffer yearly from an ICH in Catalonia. Literature comparison remains difficult since there is a lack of ICH incidence studies in populations with high multimorbidity and complexity, such as the GMA-4. In our study area, two studies conducted with complex chronic patients [14,15] showed a greater incidence of ICH. 

Similarly to the general population, GMA-4 subjects who suffered an ICH had a higher prevalence of previous cardiovascular disease, especially stroke or transient ischemic attack [25]. In men, the higher prevalence of cardiovascular risk factors and high-risk alcohol consumption translates into a higher ICH incidence density than in women. Notably, a high percentage of GMA-4 subjects show good hypertension control, which contrasts with a possible excessive prescription of drugs that increase the risk of bleeding, such as antiplatelet agents, NOACs, NSAIDs and SSRIs. These results corroborate studies that indicate a decreasing trend in hypertension-associated ICH in the last decades [26]. 

According to our results, high complexity, age ≥ 80 years and the use of antiplatelet agents independently increase the risk of ICH. The literature agrees that the incidence of ICH increases strongly with age, with an almost ten-fold increase in the yearly risk of intracerebral haemorrhage in people 85 and over compared to persons aged < 55 years [2,27,28]. However, the relationship of ICH with complexity has not yet been adequately documented. Complexity remains an ill-defined concept that can benefit from the GMA stratification, which takes into account the risk of hospital admission, mortality, visits to primary care and pharmacy expenditure.

As expected, ICH in GMA-4 patients was associated with higher mortality [29]. Although highly complex patients presented higher overall mortality when compared to the low-complexity subgroup, no differences in mortality were observed when an ICH event occurred. These results emphasise the relevance of preventive measures, particularly the identification and control of predisposing modifiable factors.

Among the risk factors identified, drugs that increase the risk of bleeding (especially antiplatelet agents) are the only modifiable factor. The high prescription of antiplatelet agents amongst GMA-4 patients responds to secondary prevention in a population with a high incidence of cardiovascular disease. Surprisingly, 42.3% of GMA-4 subjects without previous cardiovascular disease were also prescribed antiplatelet agents. Despite raising ICH risk, low-dose aspirin is one of the most widely used agents in the prevention of cardiovascular disease [30,31]. With potential benefits occasionally offset by an increased risk of bleeding, most clinical practice guidelines recommend personalised prescription of these medicines [32,33,34]. Moreover, some authors already oppose the prescription of aspirin as a primary prevention strategy in people over 70 years of age [35,36]. The concomitant use of low-dose aspirin and NSAIDs agents is also common, particularly in the elderly suffering from cardiovascular disease and pain. NSAIDs interfere with the antiplatelet effect of aspirin through competitive binding with COX-1, thus increasing the risk of ICH [37,38]. SSRIs are well established as first-line treatment for old-age depression due to their safety profile. However, by reducing serotonin levels in platelets and thus reducing platelet aggregation [39], all SSRIs have been associated with increased risk of bleeding, especially in combination with NSAIDs, aspirin, warfarin and other anticoagulants [40,41]. It is crucial that physicians consider the risk of SSRI-induced haemorrhages given the frequency and severity of depressive disorders in late life, especially in elderly patients also treated with antiplatelet agents. Finally, warfarin is the most frequent oral anticoagulant associated with ICH. Consequently, NOACs are recommended over vitamin K antagonists for the prevention of stroke in patients with AF [10]. The recent increase in anticoagulant prescriptions related to improvements in AF management might impact the incidence of ICH in the near future [42].

Our results underscore the need to incorporate a bleeding risk assessment in the GMA-4 follow up, including the risk–benefit of deprescription of drugs that increase the risk of bleeding. Our results validate changes found in the most recent review of the STOP/START criteria [43] used for deprescription in the elderly. For instance, advising against the combination of SSRIs with anticoagulant/antiplatelet agents or NSAIDs, as well as aspirin, in patients with a HAS-BLED score ≥3. Previous studies found that the HAS-BLED score could be an ICH predictor in highly complex patients [15], but we did not obtain statistically significant corroborating results in our investigation. However, a HAS-BLED ≥3 identified patients at ICH risk within GMA-4 subjects with the highest complexity (level 5), and study patients with a HAS-BLED ≥3 presented a higher ICH ID than patients with a score <3. The HAS-BLED score could support the deprescription of antiplatelet agents, NOACs, NSAIDs and SSRIs, which are highly prescribed in the study population.

Major strengths of this study are a large number of participants, the long follow-up period and the use of a stratification model developed with data from the Spanish health systems that can be easily implemented in the daily practice of family doctors. Further research is needed to develop tools to assess ICH risk and deprescription of drugs that increase the risk of bleeding in the GMA-4 population. The main limitations of the study are as follows: (1) GMAs only apply to patients who have received medical care in Primary Care, although ≥92% of the population in the study area have an active clinical record in Primary Care; (2) neither the aetiology nor the severity of ICH was differentiated due to the type of data extraction; however, it is not considered a decisive factor in the identification of risk factors, nor does it affect the final recommendations; (3) the impact of comorbidities on quality of life was not considered; (4) mortality was general and not necessarily related to ICH. 

## 5. Conclusions

GMA-4 patients present a high risk of intracerebral haemorrhage compared to the elderly and general population. In these patients, the independent risk factors associated with intracerebral haemorrhage were age ≥80 years, high complexity and the use of antiplatelet agents. The risk of bleeding must be strictly monitored in GMA-4 patients taking drugs that increase the risk of bleeding.

## Figures and Tables

**Table 1:** Baseline characteristics of GMA-4 population according to ICH occurrence during follow-up.

	Total (N = 4686)	Without ICH (N = 4516)	With ICH (N = 170)	*p* *
**Socio-demographic**				
Age (years)	84 (75–90)	84 (75–90)	84.5 (79–90)	0.082
≥65 years	4358 (93.0)	4195 (92.9)	163 (95.9)	0.178
≥80 years	3013 (64.3)	2889 (64.0)	124 (72.9)	0.021
Sex (female)	2680 (57.2)	2589 (57.3)	91 (53.5)	0.366
Institutionalised	219 (4.7)	216 (4.8)	3 (1.8)	0.100
**Cardiovascular risk factors**				
Hypertension	3828 (81.7)	3685 (81.6)	143 (84.1)	0.464
Diabetes mellitus	2048 (43.7)	1974 (43.7)	74 (43.5)	1.000
Hypercholesterolemia	2823 (60.2)	2728 (60.4)	95 (55.9)	0.270
**Comorbidities**				
Cardiovascular disease	1439 (30.7)	1372 (30.4)	67 (39.4)	0.015
Coronary artery disease	932 (19.9)	891 (19.7)	41 (24.1)	0.190
Stroke or transient ischemic attack	325 (6.9)	305 (6.8)	20 (11.8)	0.018
Peripheral artery disease	379 (8.1)	362 (8.1)	17 (10.0)	0.431
Atrial fibrillation	1053 (22.5)	1008 (22.3)	45 (26.5)	0.238
Heart failure	910 (19.4)	875 (19.4)	35 (20.6)	0.769
Thromboembolism	371 (7.9)	360 (8)	11 (6.5)	0.571
Chronic kidney disease	1072 (22.9)	1030 (22.8)	42 (24.7)	0.627
Chronic liver disease	370 (7.9)	357 (7.9)	13 (7.7)	1.000
Record of previous falls	395 (8.4)	377 (8.4)	18 (10.6)	0.373
Cognitive impairment/dementia	478 (10.2)	457 (10.1)	21 (12.4)	0.415
**Toxics**				
High-risk alcohol consumption	863 (18.4)	833 (18.4)	30 (17.6)	0.871
Smoking	838 (17.9)	809 (17.9)	29 (17.1)	0.854
**Clinical data**				
Systolic blood pressure (mmHg)	134 (125–140)	134 (125–140)	135 (130–142)	0.016
Systolic blood pressure ≥ 160 mmHg	121 (3.5)	117 (3.5)	4 (3.2)	1.000
Glycosylated haemoglobin A1c (%)	6.2 (5.6–7.1)	6.2 (5.6–7.0)	6.4 (5.5–7.4)	0.212
HAS-BLED score				0.149
0	7 (0.2)	7 (0.2)	0 (0.0)	
1	251 (7.3)	248 (7.5)	3 (2.4)	
2	1653 (48.2)	1592 (48.2)	61 (48.8)	
3	1182 (34.5)	1139 (34.5)	43 (34.4)	
4	304 (8.9)	287 (8.7)	17 (13.6)	
≥5	29 (0.9)	28 (0.9)	1 (0.8)	
**Medication**				
Oral anticoagulant (VKA/NOACs)	1117 (23.8)	1069 (23.7)	48 (28.2)	0.201
VKA	989 (21.2)	951 (21.1)	38 (22.4)	0.756
NOACs	128 (2.7)	118 (2.6)	10 (5.9)	0.025
Antiplatelet agents	2593 (55.3)	2480 (54.9)	113 (66.5)	0.004
NSAIDs	3814 (81.4)	3675 (81.4)	139 (81.8)	0.978
Statins	3110 (66.4)	2993 (66.3)	117 (68.8)	0.543
SSRIs	1702 (36.3)	1631 (36.1)	71 (41.8)	0.115
PPI	4232 (90.3)	4080 (90.3)	152 (89.4)	0.786
**GMA Multimorbidity**				
3 chronic diseases	179 (3.8)	173 (3.8)	6 (3.5)	1.000
≥4 chronic diseases	4507 (96.2)	4343 (96.2)	164 (96.5)	
**GMA Complexity**				
Low complexity (levels 2–3)	3578 (76.4)	3463 (76.7)	115 (67.7)	0.037
High complexity (levels 4–5)	1108 (23.6)	1053 (23.4)	55 (32.4)	

Data are presented as frequencies (%) or median (Q1–Q3), according to the type of variable. (*) The *p*-value corresponds to the differences in proportions using χ^2^ test for qualitative variables and Mann–Whitney U non-parametric test for continuous variables. GMA—Spanish acronym for Adjusted Morbidity Group; ICH—intracerebral haemorrhage; NOACs—new oral anticoagulants; NSAIDs—non-steroidal anti-inflammatory drug; PPI—Proton pump inhibitor; SSRIs—selective serotonin reuptake inhibitors; VKA—vitamin K antagonist.

**Table 2:** Sex differences in GMA-4 population who suffered an ICH during follow-up.

	Women (N = 91)	Men (N = 79)	*p* *
**Socio-demographic**			
Age (years)	87 (82–92)	82 (77–88)	<0.001
≥65 years	89 (97.8)	74 (93.7)	0.252
≥80 years	72 (79.1)	52 (65.8)	0.076
Institutionalised	1 (1.1)	2 (2.5)	0.598
**Cardiovascular risk factors**			
Hypertension	74 (81.3)	69 (87.3)	0.389
Diabetes mellitus	32 (35.2)	42 (53.2)	0.027
Hypercholesterolemia	58 (63.7)	37 (46.8)	0.040
**Comorbidities**			
Cardiovascular disease	29 (31.9)	38 (48.1)	0.045
Coronary artery disease	18 (19.8)	23 (29.1)	0.215
Stroke or transient ischemic attack	9 (9.9)	11 (13.9)	0.565
Peripheral artery disease	5 (5.5)	12 (15.2)	0.065
Atrial fibrillation	25 (27.2)	20 (25.3)	0.886
Heart failure	17 (18.7)	875 (19.4)	0.769
Thromboembolism	6 (6.6)	5 (6.3)	1.000
Chronic kidney disease	20 (22.0)	22 (27.8)	0.480
Chronic liver disease	4 (4.4)	9 (11.4)	0.155
Record of previous falls	12 (13.2)	6 (7.6)	0.351
Cognitive impairment/dementia	13 (14.3)	8 (10.1)	0.556
**Toxics**			
High-risk alcohol consumption	3 (3.3)	27 (34.2)	<0.001
Smoking	4 (4.4)	25 (31.6)	<0.001
**Clinical data**			
Systolic blood pressure (mmHg)	135 (129–140)	136 (130–144)	0.140
Systolic blood pressure ≥160 mmHg	1 (1.6)	3 (4.9)	0.357
Glycosylated haemoglobin A1c (%)	6.2 (5.4–7.2)	7 (5.6–7.8)	0.032
HAS-BLED score			0.001
1	2 (3.1)	1 (1.6)	
2	41 (64.1)	20 (32.8)	
3	18 (28.1)	25 (41.0)	
4	3 (4.7)	14 (23.0)	
≥5	0 (0.0)	1 (1.6)	
**Medication**			
Oral anticoagulant (VKA/NOACs)	22 (24.2)	26 (32.9)	0.275
VKA	19 (20.9)	19 (24.1)	0.756
NOACs	3 (3.3)	7 (8.9)	0.191
Antiplatelet agents	59 (64.8)	54 (68.4)	0.748
NSAIDs	74 (81.3)	65 (82.3)	1.000
Statins	66 (72.5)	51 (64.6)	0.341
SSRIs	45 (49.5)	26 (32.9)	0.043
PPI	80 (87.9)	72 (91.1)	0.666
**GMA Multimorbidity**			
3 chronic diseases	2 (2.2)	4 (5.1)	0.418
≥ 4 chronic diseases	89 (97.8)	75 (94.9)	
**GMA Complexity**			
Low complexity (levels 2–3)	64 (70.3)	51 (64.6)	0.291
High complexity (levels 4–5)	27 (29.7)	28 (35.4)	

Data are presented as frequencies (%) or median (Q1–Q3), according to the type of variable. (*) The *p*-value corresponds to the differences in proportions using χ2 test for qualitative variables and Mann–Whitney U non-parametric test for continuous variables. GMA—Spanish acronym for Adjusted Morbidity Group; ICH—intracerebral haemorrhage; NOACs—new oral anticoagulants; NSAIDs—non-steroidal anti-inflammatory drug; PPI—Proton pump inhibitor; SSRIs—selective serotonin reuptake inhibitors; VKA—vitamin K antagonist.

**Table 3:** Baseline characteristics of GMA-4 population according to complexity level.

	High complexity (N = 1108)	Low complexity (N = 3578)	*p* *
**ICH**	55 (5.0)	115 (3.2)	0.009
**Socio-demographic**			
Age (years)	86 (78–91)	84 (75–90)	<0.001
≥65 years	1055 (95.2)	3303 (92.3)	0.001
≥80 years	785 (70.8)	2228 (62.3)	<0.001
Sex (female)	571 (51.5)	2109 (58.9)	<0.001
Institutionalised	67 (6.1)	152 (4.3)	0.017
**Cardiovascular risk factors**			
Hypertension	936 (84.5)	2892 (80.8)	0.007
Diabetes mellitus	578 (52.2)	1470 (41.1)	<0.001
Hypercholesterolemia	672 (60.6)	2151 (60.1)	0.778
**Comorbidities**			
Cardiovascular disease	515 (46.5)	924 (25.8)	<0.001
Coronary artery disease	372 (33.6)	560 (15.7)	<0.001
Stroke or transient ischemic attack	111 (10.0)	214 (6.0)	<0.001
Peripheral artery disease	145 (13.1)	234 (6.5)	<0.001
Atrial fibrillation	382 (34.5)	671 (18.8)	<0.001
Heart failure	418 (37.7)	492 (13.8)	<0.001
Thromboembolism	112 (10.1)	259 (7.2)	0.002
Chronic kidney disease	95 (8.6)	275 (7.7)	0.371
Chronic liver disease	381 (34.4)	691 (19.3)	<0.001
Record of previous falls	121 (10.9)	274 (7.7)	0.001
Cognitive impairment/dementia	133 (12.0)	345 (9.6)	0.027
**Toxics**			
High-risk alcohol consumption	189 (17.1)	674 (18.8)	0.197
Smoking	204 (18.4)	634 (17.7)	0.631
**Clinical data**			
Systolic blood pressure (mmHg)	133 (124–140)	134 (126–140)	0.467
Systolic blood pressure ≥160 mmHg	43 (5.1)	78 (3.0)	0.005
Glycosylated haemoglobin A1c (%)	6.2 (5.6–7.1)	6.2 (5.6–7.0)	0.098
HAS-BLED score			<0.001
0	1 (0.1)	6 (0.2)	
1	41 (4.9)	210 (8.1)	
2	338 (40.3)	1315 (50.8)	
3	336 (40.1)	846 (32.7)	
4	110 (13.1)	194 (7.5)	
≥5	12 (1.4)	17 (0.7)	
**Medication**			
Oral anticoagulant (VKA/NOACs)	412 (37.2)	705 (19.7)	<0.001
VKA	369 (33.3)	620 (17.3)	<0.001
NOACs	43 (3.9)	85 (2.4)	0.010
Antiplatelet agents	702 (63.4)	1891 (52.9)	<0.001
NSAIDs	859 (77.5)	2955 (82.6)	<0.001
Statins	807 (72.8)	2303 (64.4)	<0.001
SSRIs	401 (36.2)	1301 (36.4)	0.947
PPI	1010 (91.2)	3222 (90.1)	0.304

Data are presented as frequencies (%) or median (Q1–Q3), according to the type of variable. (*) The *p*-value corresponds to the differences in proportions using χ2 test for qualitative variables and Mann–Whitney U non-parametric test for continuous variables. GMA—Spanish acronym for Adjusted Morbidity Group; ICH—intracerebral haemorrhage; NOACs—new oral anticoagulants; NSAIDs—non-steroidal anti-inflammatory drug; PPI—Proton pump inhibitor; SSRIs—selective serotonin reuptake inhibitors; VKA—vitamin K antagonist.

**Table 4:** ICH incidence density by age groups in the high-complexity GMA-4 population and by HAS-BLED score.

Population Considered	GMA-4 (*n*)	ICH Episodes (*n*)	ICH-ID (10,000 Person–Years)
**Total**	4686	170	85.8 (85.4–86.2)
**High complexity**	1108	55	129.0 (128.6–129.3)
<65 years	53	3	125.8 (124.3–127.2)
65–74 years	140	7	109.9 (109.1–110.7)
75–84 years	308	16	123.8 (123.2–124.4)
≥85 years	607	29	138.3 (137.8–138.8)
**HAS-BLED**			
<3	1681	64	78.1 (77.9–78.3)
≥3	1515	61	95.1 (94.8–95.3)

**Table 5:** ICH risk factors observed in the GMA-4 population according to multivariate-adjusted model.

	OR	95% CI	*p*-Value
Sex (men)	1.22	(0.88–1.69)	0.224
Age (≥80 years)	1.46	(1.03–2.08)	0.033
Stroke or transient ischemic attack	1.51	(0.92–2.46)	0.100
Antiplatelet agents	1.47	(1.06–2.05)	0.022
SSRIs	1.35	(0.98–1.87)	0.069
High GMA-complexity	1.42	(1.02–1.99)	0.037

Data are presented as odds ratio (OR) and confidence interval (95%CI). The results of the multivariate analysis are shown based on the model that was selected (according to the Akaike Information Criterion) from several models that included the following variables: sex, age (categorised ≥ 80 years), GMA complexity (categorised low (levels 2–3) and high (levels 4–5)), systolic blood pressure ≥160 mmHg, cardiovascular disease, chronic liver disease, chronic kidney disease and use of pharmacological treatment (vitamin K antagonists, non-vitamin K antagonist oral anticoagulants, antiplatelet agents, non-steroidal anti-inflammatory drug and selective serotonin reuptake inhibitors (SSRIs)). GMA—Spanish acronym for Adjusted Morbidity Group; ICH—intracerebral haemorrhage.

## Data Availability

All data are considered confidential and treated according to Regulation 2016/679 of 27 April of the European Parliament and Council on Data Protection, and the Spanish Organic Law 3/2018 of 5 December, on the protection of personal data and guarantee of digital rights. Access to the data is restricted to the research team by a password. The full dataset and statistical code are available from the corresponding author on reasonable request (jlclua@telefonica.net).

## Supplementary Materials

The following are available online at https://www.mdpi.com/article/10.3390/ijerph182413320/s1. Supplementary S1: Adjusted Morbidity Groups (GMA). Supplementary S2: Exclusion of patients with cancer. References [16,17,18,21,44,45,46,47] are cited in the supplementary materials.

## Abbreviations

AIC: Akaike Information Criterion; ATC: Anatomical Therapeutic Chemical Classification System; CI: Confidence interval; e- SAP: Electronic clinical records in primary care; GMA: Adjusted Morbidity Group (Spanish acronym for *Grupos de Morbilidad Ajustada*); HbA1c: Glycated haemoglobin; HR: Hazard ratio; ICD-10: International Classification of Diseases; ICH: Intracerebral haemorrhage; ID: Incidence density; IQR: Interquartile range; INR: International normalised ratio; MACA: Advanced Chronic Care Model (Catalan acronym for *Model d’Atenció a la Cronicitat Avançada*); NOACs: New oral anticoagulants; NSAIDs: Non-steroidal anti-inflammatory drugs; OR: Odds ratio; PPI: Proton pump inhibitor; SBP: Systolic blood pressure; SIRE: Integrated Electronic Prescription System, Catalan acronym for Sistema Integrat de Recepta Electrònica; SSRIs: selective serotonin reuptake inhibitors; VKA: vitamin K antagonist.
