# Supplementary material for: Adjusted Morbidity Groups and Intracerebral Haemorrhage: A Retrospective Primary Care Cohort Study
Source: Int J Environ Res Public Health. 2021 Dec 17;18(24):13320. doi: 10.3390/ijerph182413320 (PMC8702076; doi:10.3390/ijerph182413320)
Supplement: Supplementary file 1 [file ijerph-18-13320-s001.zip › ijerph-1472183-supplementary.pdf]

__Supplementary Materials__

Adjusted Morbidity Groups and Intracerebral Haemorrhage: A Retrospective Primary Care Cohort Study

Blanca Lorman-Carbó, Josep Lluis Clua-Espuny, Eulalia Muria-Subirats, Juan Ballesta-Ors, Maria Antònia González-Henares, Meritxell Pallejà-Millán and Francisco M. Martín-Luján

This appendix aims to provide additional information about the study.

Appendix A - Adjusted Morbidity Groups

Risk stratification has been used for healthcare transformation towards a personalized care in Catalonia, Spain [1]. Initially, the clinical risk groups (CRGs) by 3M enterprise [2] were used for population stratification to help clinicians identify people at risk of hospitalization, readmission, or death. Eventually, the CRGs evolved into Adjusted Morbidity Groups (GMA, Spanish acronym for *Grupos de Morbilidad Ajustada*) [3], a novel and country-specific tool for population grouping and risk stratification developed with data from the Spanish health system. The main motivation was the inability of the CRGs to capture the patient's complexity from a social point of view. The GMA introduced social care data and social determinants of health to refine the "complexity" concept. After testing and deployment in Catalonia, the GMA system has been implemented in 13 out of 17 Spanish regions, which means that more than 80% (38 million) of the Spanish population is currently stratified with GMAs [5]. Furthermore, it is currently being used by the Spanish Ministry of Health for their chronic care strategy [4].

The GMA classifies the population into 6 morbidity groups (GM, Spanish acronym for *Grupos de Morbilidad*) based on diagnostic codes: (1) Healthy population (GM = '00'); (2) Pregnancy and / or childbirth (GM = '20'); (3) Acute disease (GM = '10'); (4) Chronic disease in 1 system (GM = '31'); (5) Chronic disease in 2 or 3 systems (GM = '32'); (6) Chronic disease in 4 or more systems (GM = '33'); and (7) Cancer in the period of study (GM = '40'). Within each morbidity group (excluding the healthy population group), 4 cut-off points from the 40th, 70th, 85th, and 95th percentiles are identified. This generates 5 levels of complexity, which take into account risk of hospital admission, mortality, visits to primary care and pharmacy expenditure for each diagnostic code. Consequently, the population is divided into 31 mutually exclusive categories, each with a GMA code consisting of three digits that combine the morbidity group 'GM' with the level of complexity 'C' (1 to 5): GMA = 'GM' + 'C'. This process requires: (1) the diagnostic codes of morbidity; (2) date of diagnosis; and (3) the electronic primary health care records as the principal source of information. Acute diagnoses are taken into consideration only if they happened during the study period. Chronic diagnoses are considered regardless of the date.

The stratification of chronic patients in different risk levels aims to identify patients with a higher number of comorbidities, polypharmacy and higher use of services, greater risk of complications, loss of functional capacity, quality of life and/or early death. The incorporation into primary care electronic medical records of this decision-making aid is based on the premise that the family doctor determines a personalized care plan based on the risk level assigned, and the knowledge of the patient and their context [6].

In summary, the GMA allow:

- To classify the population in unique groups of morbidity (healthy population, pregnancy and childbirth, acute disease, chronic disease in 1, 2-3, or 4 systems, and active cancer). Each group is further divided into 5 levels of complexity.
- To stratify the population, assigning a unique value of complexity to each individual. This value reflects the care needs of the patient.
- To identify people suffering from specific health problems (for example, diabetes, cancer, hypertension, arthritis, or depression) in order to closely follow-up patients with greater healthcare complexity.

Appendix B

ICH is a common emergency in patients with cancer, occurring in up to 20% of patients with brain metastases [7]. ICH in cancer patients is usually caused by intratumoral haemorrhage or coagulopathy, whereas hypertensive haemorrhage is rare [8]. However, beyond the influence of primary tumour diagnosis, factors associated with risk of intracranial haemorrhage in cancer patients are largely unknown. In consequence, all cancer patients (patients with a history of cancer or with active cancer) were excluded from the study. Importantly, during GMA stratification active oncological disease has a specific morbidity group (GM = '40') and is excluded from the GMA-4 group (Appendix A). Patients with a history of cancer were excluded from the study to avoid confounders.

References

1. Bases conceptuals i model d'atenció per a les persones fràgils, amb cronicitat complexa (PCC) o avançada (MACA). 2020. Generalitat de Catalunya. Departament de Salut. Available online: https://salutweb.gencat.cat/web/.content/_ambits-actuacio/Linies-dactuacio/Estrategies-de-salut/Cronicitat/Documentacio-cronicitat/arxius/Model-de-Bases-de-Cronicitat.pdf (accessed on 20 June 2021).
2. Health Information Systems. 3M™ Clinical Risk Groups: Measuring risk, managing care. 2012. Available online: https://multimedia.3m.com/mws/media/765833O/3m-crgs-measuring-risk-managing-care-white-paper.pdf (accessed on 20 June 2021)
3. Monterde D, Vela E, Clèries M; grupo colaborativo GMA. Los grupos de morbilidad ajustados: nuevo agrupador de morbilidad poblacional de utilidad en el ámbito de la atención primaria [Adjusted morbidity groups: A new multiple morbidity measurement of use in Primary Care]. *Aten Primaria* **2016**, *48*, 674-682. doi:10.1016/j.aprim.2016.06.003
4. White Paper on Deployment of Stratification Methods. 2016. Available online: http://assehs.eu/upload/docpublicos/20/assehs_executive-summary_en.pdf (accessed on 11 May 2021).
5. Informe del proyecto de Estratificación de la Población por Grupos de Morbilidad Ajustados (GMA) en el Sistema Nacional de Salud (2014-2016). Available online: https://www.mscbs.gob.es/organizacion/sns/planCalidadSNS/pdf/informeEstratificacionGMASNS_2014-2016.pdf (accessed on 11 May 2021).
6. González González AI, Miquel Gómez AM, Rodríguez Morales D, et al. Concordancia y utilidad de un sistema de estratificación para la toma de decisiones clínicas [Concordance and usefulness of a stratification system for clinical decision making]. *Aten Primaria* **2017**, *49*, 240-247. doi:10.1016/j.aprim.2016.04.009
7. Weinstock MJ, Uhlmann EJ, Zwicker JI. Intracranial hemorrhage in cancer patients treated with anticoagulation. *Thromb Res* **2016**, *140* (Suppl. 1), S60–S65. doi:10.1016/S0049-3848(16)30100-1.
8. Velander AJ, DeAngelis LM, Navi BB. Intracranial hemorrhage in patients with cancer. *Curr Atheroscler Rep* **2012** *14*, 373-381. doi:10.1007/s11883-012-0250-3.
